# Complete Nucleotide Sequence of Australian *Tomato spotted wilt virus* Isolate TSWV-QLD2

**DOI:** 10.1128/genomeA.01267-17

**Published:** 2017-11-22

**Authors:** Richard L. Moyle, Lara Pretorius, Lilia C. Carvalhais, Peer M. Schenk

**Affiliations:** Nexgen Plants Pty. Ltd., School of Agriculture and Food Sciences, The University of Queensland, Brisbane, Queensland, Australia

## Abstract

The complete nucleotide (nt) sequence of an Australian isolate of *Tomato spotted wilt virus* was determined by deep RNA sequencing and deep small RNA sequencing. The tripartite genome consists of an 8,914-nt L segment, a 4,851-nt M segment, and a 2,987-nt S segment.

## GENOME ANNOUNCEMENT

*Tomato spotted wilt virus* (TSWV) is a member of the *Tospovirus* genus, which is the only plant-infecting genus of the *Bunyaviridae* family (1). The TSWV RNA genome is tripartite, consisting of an L segment (~8.9 kb), an M segment (~4.8 kb), and an S segment (~3 kb) (2). The M and S segments encode proteins in an ambisense orientation with overlapping noncoding intergenic regions (2, 3). TSWV is vectored by thrips in a circulative propagative manner (2). TSWV has an extensive host range, including many crop, weed, landscape, and native plants (2, 4). TSWV infection symptoms can vary between cultivars and species but may include stunting, wilting, chlorosis, necrosis, and the formation of chlorotic or necrotic spotting and rings (1).

A large number of partial TSWV sequences have been lodged in the GenBank database, with many N protein-coding sequences identified from a range of geographic locations. Far fewer TSWV genomes have been fully sequenced. Recently, the first fully sequenced genome of an Australian TSWV isolate was reported (5). Here, we report the complete nucleotide (nt) sequence of a second Australian TSWV isolate, designated TSWV-QLD2.

The TSWV-QLD2 isolate was collected from a commercial crop of *Capsicum annuum* cv. Warlock from the Bowen region of Queensland, Australia, in 2015. The isolate was maintained in *Capsicum annuum* cv. California Wonder and later inoculated into tomato cv. Moneymaker. Total RNA was isolated from systemically infected tomato plants approximately 30 days postinoculation, using previously described methods (6). The RNA was subjected to both deep RNA sequencing and deep small RNA sequencing using Novogene as the sequencing service provider. After quality trimming, the RNA sequencing reads were assembled to the TSWV-QLD1 reference genome using Geneious version 8.1.7 software, as previously described (7). Similarly, after quality trimming and selection of a 20- to 24-nt subset, the small RNA sequencing reads were assembled to the TSWV-QLD1 reference genome using Geneious version 8.1.7 software, as previously described (5). Both RNA sequencing and small RNA sequencing methods generated identical full coverage genome sequences.

The TSWV-QLD2 isolate is present as a quasi-species. Within the 8,914-nt L segment, four clear polymorphisms were identified. Two polymorphisms were identified within the 4,851-nt M segment sequence, while the 2,987-nt S segment contained three polymorphisms.

Phylogenetic analysis was performed using previously described methods (5, 8). TSWV-QLD2 shares the highest percentage identity with the previously reported TSWV-QLD1 genome sequence (98 to 99% between the L, M, and S segments). TSWV-QLD2 shares sequence identities of between 93 and 97% for the L segment and 91 to 96% for the S and M segments of other fully sequenced TSWV isolates from diverse geographic locations. The N protein sequence encoded by the TSWV-QLD2 isolate is identical to that encoded by the previously described TSWV-QLD1 isolate and therefore clustered within the same clade (5).

### Accession number(s).

The GenBank accession numbers for the TSWV-QLD2 L, M, and S segments are MG025802, MG025803, and MG025804, respectively.
